# Biochemical Composition of Pumpkin Seeds and Seed By-Products

**DOI:** 10.3390/plants13172395

**Published:** 2024-08-27

**Authors:** Nikolaos Polyzos, Ângela Fernandes, Ricardo C. Calhelha, Jovana Petrović, Marina Soković, Isabel C. F. R. Ferreira, Lillian Barros, Spyridon A. Petropoulos

**Affiliations:** 1 Laboratory of Vegetable Production, Department of Agriculture, Crop Production and Rural Environment, University of Thessaly, Fytokou Street, 384 46 Volos, Greece; npolyzos@uth.gr; 2 Centro de Investigação de Montanha (CIMO), Instituto Politécnico de Bragança, Campus de Santa Apolónia, 5300-253 Bragança, Portugal; afeitor@ipb.pt (Â.F.); calhelha@ipb.pt (R.C.C.); iferreira@ipb.pt (I.C.F.R.F.); 3 Laboratório Associado para a Sustentabilidade e Tecnologia em Regiões de Montanha (SusTEC), Instituto Politécnico de Bragança, Campus de Santa Apolónia, 5300-253 Bragança, Portugal; 4 Institute for Biological Research “Siniša Stanković”—National Institute of Republic of Serbia, University of Belgrade, Bulevar despota Stefana 142, 11108 Belgrade, Serbia; jovana0303@ibiss.bg.ac.rs (J.P.); mris@ibiss.bg.ac.rs (M.S.)

**Keywords:** *Cucurbita maxima* L., seed oils, proximate composition, seed cakes, bioactive properties, fatty acids composition

## Abstract

The goal of the current work was to assess the nutritional profile and phytochemical properties of cucurbit (*Cucurbita maxima* L.) seeds, seed oils and oil extraction by-products (e.g., seed-cakes). Our results suggest a high nutritional value for both cucurbit seeds and cucurbit cake, while γ-tocopherol was the richest compound, with traces of α, β and δ-tocopherol compounds also detected. Regarding the free sugars composition, there were recorded significant statistical differences between seeds and cucurbit seed-cake, although sucrose content was the highest for both matrices (1.97 and 2.9 g/100 g dw, respectively) followed by trehalose (0.26 and 0.25 g/100 g dw, respectively), fructose (0.20 and 0.34 g/100 g dw, respectively) and glucose (0.21 and 0.19 g/100 g dw, respectively). In terms of organic acids, oxalic was the only compound detected in seed cake (0.006 g/100 g dw), while in seeds only traces of oxalic and malic acid were detected. In relation to fatty acid composition, linolenic acid was the most abundant compound in both seeds and seed-cake (43.9% and 41.5%, respectively), while oleic acid (37.0% and 36.3%, respectively), palmitic acid (12.2% and 14.0%, respectively) and stearic acid (4.83% and 5.46%, respectively) were detected in lesser amounts. Moreover, polyunsaturated fatty acids (PUFA) were the major fatty acids class (44.5% and 42.3% in seeds and seed cake, respectively) compared to monounsaturated fatty acids (MUFA; 37.4% and 36.7% in seeds and seed cake, respectively) and saturated fatty acids (SFA; 18.1% and 21.0% in seeds and seed cake, respectively) which were detected in lower amounts. Furthermore, the tested extracts did not present any cytotoxic or hepatoxic activity at the maximum tested concentration (GI_50_ > 400 μg/mL), while seed oils presented satisfactory antimicrobial properties with inhibitory activity against the studied bacterial strains and fungi. Our findings provide valuable knowledge regarding the exploitation of pumpkin seeds and seed by-products as valuable natural sources of nutrients and phytochemicals in the food industry sector within the context of a circular economy.

## 1. Introduction

Pumpkin is a vegetable with functional properties belonging to the Cucurbitaceae family which includes 130 genera and 800 species, while the most commonly used species for commercial cultivation worldwide are *Cucurbita maxima* L., *C. pepo* L. and *C. moschata* Duchesne [1]. The global production of pumpkin in 2018 surpassed 27.6 million tons from an area of 2.04 million hectares including squash and gourd, while for 2022 the respective values were 22.8 million tons and 1.5 million hectares [2,3]. Common cultivated pumpkin fruits have thicker rind and more dense color but also have less fibrous flesh compared to the wild species; however, fruit characteristics are highly variable among the species and cultivars in terms of shape, size and color depending on the genotype and climate conditions [4]. The consumption of pumpkins has been known since a long time ago, being significant in human diet especially in countries where their food use is significantly increased because of their widespread cultivation [5]. Nowadays, pumpkin is acknowledged as a highly consumed fruit vegetable all over the world [6]. The incorporation of pumpkin fruit into many recipes and dishes, as well as the increasing interest of consumers who seek a healthy lifestyle have highlighted the rich nutritional value and phytochemical characteristics of the species [7,8]. For instance, *C. maxima* fruit are a rich source of phenolic compounds, carotenoids and terpenoids, which are highly associated with the high antioxidant activity of the plant [9]. Similarly, pumpkin seeds are considered a valuable source of essential minerals which provide health benefits, and they protect against non-communicable disorders such as cancer, diabetes, hyperglycemia and microbial infections [10,11,12]. In the same manner, there are many reports mentioning that the extracts of pumpkin plant tissues could be exploited as an alternative to synthetic medicines against obesity, and can also act as anticancer, antidiabetic and antimicrobial agents [13,14,15].

Moreover, although seeds are usually considered a waste product, more and more researchers have shown great interest in exploiting and highlighting their nutritional value and phytochemical profile, whereas recent reports suggest that seeds could be considered as renewable resources with many products being derived from them (e.g., flour, seed meals, seed oils), which could be valorized through their incorporation in other food ingredients or as functional ingredients [16]. However, so far the lack of knowledge and technological solutions hinders the full exploitation of this valuable by-product [17]. For that reason, the utilization of waste products through the development of sustainable economic systems and the adoption of a circular economy approach in agri-business has been the main priority of the scientific community during recent years. Therefore, the goal of the present work was to investigate the nutritional and chemical composition, and the bioactivities of raw pumpkin seeds and seed cakes, as well as the chemical and bioactive properties of seed oils focusing on their potential valorization in the food and related industry sectors.

## 2. Results and Discussion

The results of nutritional analysis of seeds and seed cakes of pumpkin are presented in Table 1. As expected, raw seeds contained significantly higher amounts of fat compared to seed cakes (42.74 g/100 g dw vs. 7.62 g/100 g dw) obtained after the oil extraction process with a screwing press. On the other hand, seed cakes were richer than raw seeds in terms of protein (58.6 g/100 g dw vs. 37.7 g/100 g dw), ash (5.40 g/100 g dw vs. 3.52 g/100 g dw) and carbohydrates content (28.4 g/100 g dw vs. 16.07 g/100 g dw), whereas the energy content was higher for raw seeds mostly due to their higher lipidic content (599.6 kcal/100 g dw and 416.5 g/100 g dw, respectively). Karrar et al. [5], who determined the nutritional and antioxidant properties of different Cucurbitaceae species from Sudan, reported a proximate composition with values of fat, ash and carbohydrates within the range values as in our study, although they recorded a lower protein content in contrast to the findings of this study which could be mostly attributed to genotypic differences and to differences in the environmental conditions and cultivation conditions. In contrast, Tzortzakis et al. [9] reported similar values of proteins for *Cucurbita* seeds which are a rich source of proteins, while the detected amounts of nutrients in our study were similar to the ones suggested by Salehi et al. [18], except for protein content which varied among the studied species (e.g., *Cucurbita pepo*, *C. moschata* and *C. maxima*). Moreover, Badr et al. [19] also suggested significant differences in the nutritional profile of seed flour and defatted seed meal obtained from ripe pumpkin fruits cultivated in Egyptian habitats which had a high content of proteins (35.95 g/100 g dw and 70.15 g/100 g dw, respectively). Leichtweis et al. [20] suggested a great variability in the nutritional profile of flesh among several genotypes of pumpkin (*Cucurbita* sp.) cultivated in Greece under the same conditions as in our study, which is attributed to the high impact of genetic background, growing conditions, pre-harvest and post-harvest practices, as well as the diverse domestication pathways [9,21] and the considerable genetic heterogeneity at the intrapopulational level, due to out-crossing cross-fertility among the species (e.g., interspecific hybrids among *C. moschata* with *C. maxima* and *C. pepo*) [22]. Similarly, the rich content of protein detected in the current study indicates the high nutritional value of raw pumpkin seeds which has also been demonstrated by the research of Karanja et al. [23], who studied the nutritional composition of different pumpkin species (not indicated in the study) cultivated in Kenya. However, a varied protein content may be recorded in seed extracts depending on the extraction protocol and the yield of recovered protein [24]. Regarding the nutritional profile of seed cake, similar results have also been confirmed by Bhat and Bhat [25] who tested blended seed sake from fruit cultivated in India (the species is not indicated in the study) and Zdunczyk et al. [26] who tested seed cakes obtained from the oil pressing industry and ground seeds of *C. pepo*; however, due to the large pool of genetic variability in the genus and the hybridization among the species, it is very common to observe differences in nutritional value of pumpkin fruit parts [27].

The tocopherol profile of the studied pumpkin samples (seeds and seed cake) is described in Table 2. All the isoforms of vitamin E were detected in both matrices in varied amounts, while total tocopherols content was richer in raw seeds (Figure S1) than in the seed cake (6.96 g/100 g dw and 1.18 g/100 g dw, respectively). γ-Tocopherol was the most abundant tocopherol (6.59 g/100 g dw and 1.07 g/100 g dw in seeds and seed cake, respectively) followed by α, β and δ-tocopherol in varied amounts in seeds and seed cake. In particular, in the case of seeds, α-tocopherol was the second richest compound followed by δ- and β-tocopherol, while in seed cake β-tocopherol was detected in high amounts followed by α- and δ-tocopherols. High tocopherol content is a desirable quality parameter due to its pivotal role in protection against oxidative stress and the development of chronic diseases as well as its beneficial health effects [5]. The findings of our work are in total accordance with the results of Kim et al. [28], who also reported a high content of γ- and δ-tocopherol in *Cucurbita* species (e.g., *C. pepo*, *C. moschata*, *C. maxima*) cultivated in Korea, although the same authors did not identify β- and δ-tocopherol in the fruit parts (flesh, peel seed) of any species. By contrast, Stevenson et al. [29] indicated that the seed oil of 12 pumpkin cultivars (*C. maxima* Duchesne) cultivated in Iowa state (USA) contained large amounts of δ-tocopherol, followed by γ- and α-tocopherol, while they suggested a significant impact of the genotype on tocopherols composition. Similar results were suggested by Rezig et al. [30], who also identified δ-tocopherol as the main vitamin E isoform in the seed oil of *C. maxima* fruit, while they also detected significant amounts of both γ- and α-tocopherol. The results of the work of Rabrenović et al. [31] were in the same line as in our work, since the authors detected high content of β- and γ-tocopherol and lower content of α- and δ-tocopherol in the seed oil obtained from naked seeds and the seed husk of different pumpkin hybrids and cultivars (*C. pepo*) cultivated in Hungary or obtained from commercial products from Serbia, while similar findings were suggested by Singh et al. [32] for seeds and seed kernels of *C. moschata* varieties and Ryan et al. [33] for *Cucurbita* spp. seeds.

Regarding free sugar composition, sucrose was the richest compound in both seeds and seed cake (1.97 g/100 g dw and 2.07 g/100 g dw, respectively), followed by trehalose, glucose and fructose which were detected in lesser amounts (Table 2). Moreover, the seed cake (Figure S2) had a higher content of sucrose, fructose and total sugars, and lower amounts of glucose than seeds, while no significant differences were recorded in trehalose content. Similar findings were reported by Mansur et al. [34] who also suggested that sucrose was the main compound in dehulled pumpkin seeds (*C. pepo* Kakai 35 obtained from fruit cultivated in Hungary), whereas they detected stachyose and raffinose which were not identified in our study. Sucrose was also the main sugar identified in different pumpkin fruit parts (e.g., flesh, peel and seed) by Hagos et al. [35], whereas the same authors suggested that fructose and glucose were identified only in flesh and peel samples and not in seeds from fruit of *C. maxima* plants cultivated in four regions of Ethiopia.

Similarly, the organic acids profile varied between seeds and seed cake with oxalic acid being identified in low amounts only in seed cake, while traces of oxalic and malic acid were identified in seeds and seed cake. On the other hand, Mohaammed et al. [36] reported that *C. maxima* seeds grown in Nigeria were richer in oxalates than the flesh and bark of *C. maxima* fruit, while Singh et al. [32] reported a varied content of oxalates among the seeds of different *C. moschata* Duch. varieties grown in India. Overall, the contrasting results observed in the literature reports regarding the chemical composition of pumpkin could be justified by the large pool of genetic variability, the growing conditions and the extraction method [33,37,38].

The fatty acids profile (%) in seeds and seed by-products (seed cake and seed oil) are described in Table 3. The richest fatty acids detected were linolenic acid (C18:2n6c), oleic acid (C18:1n9c+t), palmitic acid (C16:0) and stearic acid (C18:0), whereas polyunsaturated fatty acids (PUFA) were the richest class of fatty acids. Moreover, there were significant statistical differences regarding the fatty acid profile based on the studied matrices (e.g., seeds, seed cake and seed oil). In particular, 21 individual compounds were identified in seeds (Figure S3) and seed cake, while seed oils (Figure S4) contained 13 individual fatty acids. In addition, seed oils were richer in linolenic, palmitic and stearic acids (55.5%, 14.31% and 6.22%, respectively), while seeds and seed cake contained the highest amounts of oleic acid (37.0% and 36.3%, respectively). A similar trend was reported for the content of the different classes of fatty acids, where seed oils had the highest content of PUFA and SFA, while seeds and seed cake had the highest content in MUFA.

According to the work of Seymen et al. [39], the main fatty acids in seed oils obtained from 10 pumpkin genotypes (*C. pepo*) grown in Turkey were similar to our study, although a varied profile was observed among the studied genotypes with linoleic or oleic acid having the highest content. The same fatty acids were the major ones detected in the work of Kim et al. [28], although the authors suggested a varied profile depending on the *Cucurbita* species, while Idouraine et al. [40] indicated that oleic acid was the main fatty acid in the de-hulled seeds of eight *Cucurbita pepo* lines cultivated in Mexico. In contrast, Gohari Ardabili et al. [41] detected a similar content of oleic and linoleic acid in the seed oil of *C. pepo* sbsp. *pepo* var. syriaca seeds grown in Iran, while Rezig et al. [30], Nyam et al. [42] and Montesano et al. [43] recorded a higher content of oleic compared to linoleic acid in pumpkin seed oils (*C. pepo* plants cultivated in Malaysia and *C. maxima* var. Berrettina plants grown in Italy, respectively). The findings of our work also agree with other literature data where the composition of fatty acids of seeds oils and processed or unprocessed seeds of different *Cucurbita species* were investigated [44,45,46]. In the study of Procida et al. [47], commercial pumpkin seed oils of various origins (Italy and Slovenia) exhibited a varied profile of fatty acids, thus indicating a significant impact of growing conditions, the genotype and the extraction method on the chemical profile of the recovered oil, while Mitra et al. [48] highlighted the importance of fine-tuning the extraction parameters (e.g., extraction pressure, temperature and time) in order to maximize the oil recovery and oil quality through the supercritical extraction method. Although linoleic acid is prone to oxidation, it also has multiple health beneficial effects related to the prevention of heart disease, cancer and other chronic diseases, thus suggesting the high nutritive value of pumpkin seed oils [30]. In contrast, the high amount of oleic acid in seeds and seed cake make these matrices ideal for industrial applications [29]. Furthermore, the high PUFA and MUFA content in the studied matrices suggest a health-beneficial role due to their protective effects against cardiovascular diseases, hypertension and arthritis, and positive effects on brain development and nervous system function [49].

The antiproliferative activity and hepatotoxicity of the examined extracts are shown in Table 4. According to the findings of this study, none of the studied extracts showed anti-proliferative or hepatotoxic effects (GI_50_ values higher than 400 μg/mL), apart from the seed oil which presented a mild anti-proliferative effect against the HeLa (cervical carcinoma) cell line (327 µg/mL). Similarly to our work, Tzortzakis et al. [20] suggested mild anti-proliferative properties of pumpkin seed oil against the HeLa cell line, whereas the same authors suggested a mild toxicity towards the PLP2 non-tumor cell line and lack of anti-proliferative against MCF-7, HepG2 and NCI-H460 cell lines which was also observed in our work. According to the literature, cucurbitacins that various plant parts of *Cucurbita* species may contain, especially the seeds, have been linked with mammalian poisoning on several occasions [18]. Contrarily, Chari et al. [50] highlighted the efficacy of seed extracts of *C. pepo* plants grown in India against colon cancer in Wistar rats, while Bahadori et al. [51] suggested the anti-proliferative and apoptotic effects of *C. pepo* seed extracts against cancer of the endocrine system. Vinayashree et al. [52] reported that protein fractions obtained from *Cucurbita moschata* var. Kashi Harit seeds grown in India showed promising results against different cancer cell lines (e.g., HepG2, MDA-MB-231, and MCF-7), while extracts of *Cucurbita pepo* L. subsp. *pepo* var. styriaca seeds inhibited the growth of human dermal fibroblast (HDF-5) cells [53]. Moreover, several other reports indicate the antiproliferative effects and the lack of toxicity towards non-tumor cell lines of different plant parts of *Cucurbita* species (e.g., pulp and skin of fruit or leaves) [11,20,54,55]. Contrarily, Parry et al. [53] did not observe significant cytotoxic properties for pumpkin seed flours (*C. pepo* L. cv. Triple Treat) obtained after oil extraction with a cold press against HT-29 cell lines. The contrasting results in the literature could be due to the different *Cucurbita* species tested and to differences in phytochemical content which may be affected by environmental factors and agronomic practices and by the extraction protocols [20].

The antibacterial and antifungal activity of pumpkin seed oil are presented in Table 5 and Table 6. The studied seed oil had satisfactory antibacterial effects against the tested bacteria, namely *B. cereus*, *M. flavus*, *E. cloacae*, *S.* Typhimurium and *E. coli* by showing similar or lower MIC and/or MBC values than E211 and/or E224 which were used as positive controls, especially against *M. flavus* where MIC and MBC values were comparable to both compounds. Likewise, pumpkin oil was effective against most of the tested fungi with similar or lower MIC and/or MFC values than E211 and/or E224, especially for the case of *P. verrucosum* var. Cyclopium where seed oils had lower MIC values than both positive controls, whereas seed oil was not effective against *A. fumigatus* showing higher MIC and MBC values than E211 and E224. The antimicrobial effects of pumpkin seed oil have been highlighted in several studies. Hussain et al. [56] suggested that extracts of pumpkin seed collected from Pakistan were more effective against various bacterial strains (*Streptococcus aureus*, *Bacillus subtilis*, *Escherichia coli* and *Salmonella typhi*) and fungi (e.g., *Fusarium oxysporum*, *Trichoderma* spp., *Mucor miehei* and *Candida albicans*) compared to flesh and peels, while the same oils were very effective against several fungi (e.g., *A. versicolor*, *A. niger, P. funiculosum*, *P. verrucosum* var. Cyclopium) with MIC values similar or lower than the positive controls tested (e.g., E211 and E224). Leichtweis et al. [20] also reported significant antimicrobial properties for pumpkin plant parts which differed depending on the growing location (e.g., Portugal and Algeria). Moreover, Badr et al. [19] suggested that pumpkin seed oil was effective against *Saccharomyces cerevisiae*, while defatted seed meal showed no antimicrobial properties, thus suggesting that these activities are related to compounds retrieved during oil extraction. In the work of Petropoulos et al. [57], pumpkin seed oil (*C. maxima* L. plants grown in Greece) showed important antibacterial effects against a broad range of bacterial strains with activities comparable to positive controls. Similarly to our work, Abd El-Aziz et al. [58] suggested that seed oils of *C. moschata* plants grown in Egypt were efficient against *B. subtilis*, *S. aureus*, *E. coli* and *Klebsiella pneumoniae*, as well as against the fungi *Rhodotorula rubra* and *C. albicans*, while Sener et al. [59] reported significant antibacterial effects for seeds oils of *C. pepo* plants collected from Turkey against *K. pneumoniae* and *Acinetobacter baumannii.* Contrarily, Saavedra et al. [60] did not record any antibacterial properties for squash seeds (*C. pepo* plants cultivated in Northern Portugal) against four different bacteria (e.g., *Staphylococcus aureus*, *Listeria monocytogenes, Pseudomonas aeruginosa* and *E. coli*). These contradictory results in scientific reports regarding the antimicrobial activity properties of pumpkin seeds and seed oil extracts suggest significant differences in the phytochemical content due to genetic variability, environmental and cultivation conditions, the extraction method and the plant part studied.

## 3. Materials and Methods

### 3.1. Plant Material and Growing Conditions

Pumpkin seeds from the local cultivar “Nychaki” (*Cucurbita maxima* L. cv. Nychaki) were sown directly in soil in single rows (27 July 2022) and fruit were harvested at marketable maturity (7 December 2022), i.e., fruit reached the maximum size and the characteristic skin color for the cultivar. The experiment was conducted at the experimental farm of the University of Thessaly, at Velestino, Greece (39°37′18.6″ N, 22°22′55.1″ E; altitude: approximately 120 m above sea level (asl)). Plants were distanced at 1.5 m × 2.5 m, while 100 plants in total were used (three rows 50 m long) in an experimental plot of 150 m^2^. Agronomic practices were applied as previously described by Petropoulos et al. [57]. Plants were irrigated regularly via drip irrigation (water supply of 6 L/h per emitter and per plant) based on the readings of soil moisture content through PR2 Profile Probes (Delta T PR2/4 + HH2; Delta-T Devices Ltd., Burwell, UK) [55]. After harvest, fruit were cut in halves and seeds were collected, washed with distilled water and air-dried at room temperature until they reached 10% moisture content. After collection, seeds were stored at room temperature (approximately 25 ± 1 °C) until the oil extraction took place (approximately two months after collection).

### 3.2. Seed Oil Extraction

The extraction of oil from pumpkin seeds was performed with a cold press following the protocols previously described by the authors [61]. The cold press (oil temperature was kept at 24–26 °C throughout the extraction process) used was the Oil press/expeller KK Oil prince F Universal 230V (Kern Kraft Oil press GmbH & Co. KG; Reut, Germany). Seed cake consisted of seed residues after oil extraction with the oil press.

### 3.3. Proximal Composition of Seeds and Seed Cakes of Pumpkin

The Official Methods of Analysis of the Association of Official Analytical Chemists [62] were implemented for the assessment of proximal composition (proteins, lipids, ash, carbohydrates, and total energy) of seeds and seed cakes of pumpkin samples. Protein content was assessed by the macro-Kjeldahl method (conversion factor = 6.25), via acid digestion, distillation, and titration [63]. Crude fat content was estimated after extraction for 7 h with petroleum ether in a Soxhlet apparatus, while the ash content was evaluated after incineration (550 ± 10 °C) in a muffle furnace. Total carbohydrates and energetic value were calculated according to the formulas provided by Harumi Iyda et al. [63].

### 3.4. Chemical Profile of Hydrophilic Compounds

#### Free Sugars

To determine the content in free sugars, 40 mL of ethanol solution (80%) and 1 mL of Internal Standard (melezitose, 25 mg/mL) were added to 1 g of dry and defatted samples at 80 °C for 30 min. The resulting suspension was centrifuged (Centurion K24OR refrigerated centrifuge, West Sussex, UK) at 15,000× *g* for 10 min. The supernatant was concentrated at 60 °C under reduced pressure and defatted three times with 10 mL of ethyl ether, successively. After concentration at 40 °C, the solid residues were dissolved in water to a final volume of 5 mL and filtered through 0.2 μm Whatman nylon filters [64]. The analysis was conducted using a High-Performance Liquid Chromatography (HPLC) array coupled to a refraction index detector. The equipment of analysis consisted of an integrated system with a pump (Knauer, Smartline system 1000, Berlin, Germany), degasser system (Smartline manager 5000), auto-sampler (AS-2057 Jasco, Easton, MD, USA) and an RI detector (Knauer Smartline 2300). Data were analyzed using Clarity 2.4 Software (DataApex, Prague, Czech Republic). The chromatographic separation was achieved with a Eurospher 100-5 NH2 column (4.6 × 250 mm, 5 mm, Knauer, Berlin, Germany) operating at 30 °C (7971 R Grace oven). The mobile phase was acetonitrile/deionized water, 70:30 (*v*/*v*) at a flow rate of 1 mL/min. The compounds were identified by chromatographic comparisons with authentic standards. Quantification was performed using the internal standard method and sugar contents were further expressed in g/100 g of dry weight (dw).

The organic acids were analyzed using ultra-fast liquid chromatography coupled to a photodiode array detector (UFLC–PDA). Samples (approximately 2 g) were extracted by stirring with 25 mL of meta-phosphoric acid (25 °C at 150 rpm) for 45 min and subsequently filtered through Whatman No. 4 paper [65]. The analysis was performed using a Shimadzu 20A series UFLC (Shimadzu Corporation, Kyoto, Japan). Separation was achieved on a SphereClone (Phenomenex, Torrance, CA, USA) reverse phase C18 column (5 μm, 250 mm × 4.6 mm i.d.) thermostatted at 35 °C. The elution was performed with sulfuric acid (3.6 mM) using a flow rate of 0.8 mL/min. Detection was carried out in a PDA, using 215 and 245 nm (for ascorbic acid) as preferred wavelengths. The identified organic acids were further quantified and based on the comparison of chromatographs with commercial standards.

### 3.5. Analysis of Lipophilic Compounds

#### 3.5.1. Tocopherols

The evaluation of tocopherol content was conducted with HPLC equipment (see the methodology for Section Free Sugars) and a fluorescence detector (FP-2020; Jasco, Tokyo, Japan). BHT solution in hexane (10 mg/mL; 100 μL) and IS solution in hexane (tocol; 50 μg/mL; 400 μL) were added to the sample prior to the extraction procedure. The samples (approximately 500 mg) were homogenized with methanol (4 mL) by vortex mixing (1 min). Subsequently, hexane (4 mL) was added and again vortex mixed for 1 min. After that, saturated NaCl aqueous solution (2 mL) was added, the mixture was homogenized (1 min), centrifuged (5 min, 4000× *g*) and the clear upper layer was carefully transferred to a vial. The sample was re-extracted twice with hexane. The combined extracts were taken to dryness under a nitrogen stream, redissolved in 2 mL of n-hexane, dehydrated with anhydrous sodium sulphate, filtered through 0.2 μm nylon filters from Whatman, and transferred into a dark injection vial prior to analysis, according to the method of Spréa et al. [64]. The fluorescence detector was programmed for excitation at 290 nm and emission at 330 nm. The chromatographic separation was achieved with a Polyamide II (250 mm × 4.6 mm i.d.) normal-phase column from YMC Waters operating at 30 °C. The mobile phase used was a mixture of n-hexane and ethyl acetate (70:30, *v*/*v*) at a flow rate of 1 mL/min, and the injection volume was 20 μL. The IS method was used to measure the fluorescence signal response, and chromatographic comparison with standards served as the basis for quantification.

#### 3.5.2. Analysis of Fatty Acids

The crude fat content of seeds and seed cakes of pumpkin samples obtained after Soxhlet extraction and 1 mL of crude seed oils were methylated with 5 mL of methanol/sulfuric acid 95%/toluene 2:1:1 (*v*/*v*/*v*) for at least 12 h in a bath at 50 °C and 160 rpm; in order to obtain phase separation, deionized water (3 mL) was added; the fatty acids methyl esters (FAME) were recovered by shaking in a vortex with 3 mL of diethyl ether, and the upper phase was recovered into an amber vial with anhydrous sodium sulphate to eliminate the water and filtered through a 0.2 μm Whatman nylon filter [61].

Fatty acids were determined by gas-liquid chromatography with flame ionization detection, employing a YOUNG IN Crhomass 6500 GC System instrument(KRSS Ltd., Wellingborough, UK) supplied with a split/splitless injector set at 250 °C with a split ratio of 1:80, a flame ionization detector set at 260 °C, and a Zebron-Fame column [66]. The detected compounds were identified by comparison with the peaks of fatty acid methyl ester (FAME).

### 3.6. Evaluation of Bioactive Properties In Vitro

#### 3.6.1. Oil Extracts Preparation

The studied oils (5 mL) were extracted using 10 mL of methanol over 3 repeats. Then, all the extracts were combined and dried using anhydrous sodium sulphate, after which they were filtrated and evaporated until dryness with reduced pressure.

#### 3.6.2. Hydroethanolic Extraction

The seeds and seed cakes of pumpkin samples (approximately 2 g) were stirred in 30 mL of an ethanol/water mixture (80:20 vol.) at 25 °C for 1 h and later filtered using a filter paper (Whatman No. 4). The residue was extracted with an additional 30 mL of the hydroethanolic mixture in the same conditions (80:20 vol. ethanol/water mixture at 25 °C). The combined extracts were concentrated using a Büchi R-210 rotary evaporator (Flawil, Switzerland) at 40 °C and lyophilized with a FreeZone 4.5 system (Labconco, Kansas City, MO, USA) [63].

#### 3.6.3. Anti-Proliferative Activity

The antiproliferative activity of the studied extracts was determined according to previously described protocols [64,67], using the sulforhodamine B (Sigma-Aldrich, St. Louis, MO, USA) colorimetric assay against four human tumor cell lines: i.e., HeLa (cervical carcinoma), HepG2 (hepatocellular carcinoma), MCF-7 (breast carcinoma), and NCI-H460 (non-small cell lung cancer), and a primary cell line obtained from pig liver (PLP2). Ellipticine was implemented as positive control. The results were expressed as GI_50_ values (μg/mL).

#### 3.6.4. Antimicrobial Activity Evaluation

For the antimicrobial properties of the extracts, Gram-positive bacteria (*Staphylococcus aureus* (ATCC 11632), *Bacillus cereus* (food isolate), *Micrococcus flavus* (ATCC 10240)), as well as Gram-negative bacteria (*Enterobacter cloacae* (ATCC 35030), *Escherichia coli* (ATCC 25922) and *Salmonella* Typhimurium (ATCC 13311)) were tested. For antifungal assays, the following micromycetes were used: *Aspergillus fumigatus* (ATCC 9197), *Aspergillus versicolor* (ATCC 11730), *Aspergillus niger* (ATCC 6275), *Penicillium funiculosum* (ATCC 36839), *Penicillium verrucosum* var. Cyclopium (food isolate) and *Trichoderma viride* (IAM 5061). All antimicrobial properties were assessed following the microdilution method [68].

### 3.7. Statistical Analysis

For each analysis, three biological samples were used, and all the assays were performed in triplicate. The results were expressed as mean values and standard deviation. Student’s *t*-test was applied to detect significant differences among two samples at α = 0.05. For the results of fatty acids composition and bioactive properties, a one-way analysis of variance (ANOVA) was applied, while means were compared with Tukey’s HSD test, with α = 0.05. The analysis was carried out using SPSS v. 22.0 program SPSS Statistics software (IBM Corp., Armonk, NY, USA).

## 4. Conclusions

The current study evaluated the nutritional profile, chemical profile and bioactivities of pumpkin by-products, including seeds, seed cake and seed oils, aiming to explore them as alternative sources of nutrients and phytochemicals within the circular economy context. Our results suggested significant statistical differences between the tested parts, with seeds and the seed cake being rich in proteins and tocopherols, especially γ-tocopherol. Moreover, all the studied matrices were rich in fatty acids, especially linolenic acid and oleic acid, showing a health-beneficial profile of fatty acids. None of the tested extracts showed cytotoxic effects towards non-tumor cell lines, suggesting that all of them could be introduced in human diet and contribute to overall well-being, while mild efficacy was shown against HeLa cell lines. Finally, seed oils showed promising antimicrobial properties against a broad range of bacteria and fungi, thus indicating the potential of incorporating them in food products aiming to improve their functionality and health-beneficial effects, as well as to increase their shelf-life. Therefore, our results provide novel information related to chemical characterization of seeds and seed byproducts of a local landrace of pumpkin, focusing on the potential of valorizing these by-products as alternative sources of nutrients and bioactive phytochemicals that could be exploited by the food industry through the design of healthy foods with functional properties within the context of sustainable use of natural resources.

## Figures and Tables

**Table 1:** Nutritive (g/100 g dw) and energetic value (kcal/100 g dw) of the studied pumpkin seeds and seed cake (mean ± SD).

	Fat	Proteins	Ash	Carbohydrates	Energy
Seeds	42.74 ± 0.09	37.67 ± 0.20	3.52 ± 0.09	16.07 ± 0.03	599.62 ± 0.10
Seed cake	7.62 ± 0.08	58.58 ± 0.30	5.40 ± 0.05	28.40 ± 0.20	416.50 ± 0.40
Student’s *t*-test *p*-Value	<0.001	<0.001	<0.001	<0.001	<0.001

Significant differences (*p* < 0.001) between samples were assessed by Student’s *t*-test.

**Table 2:** Tocopherols (mg/100 g dw), free sugars (g/100 g dw) and organic acids (g/100 g dw) profile of the pumpkin seeds and seed cake (mean ± SD).

	Seeds	Seed Cake	
Tocopherols (mg/100 g dw)			Student’s *t*-test *p*-Value
α-Tocopherol	0.075 ± 0.004	0.018 ± 0.001	<0.001
β-Tocopherol	0.011 ± 0.001	0.079 ± 0.002	<0.001
γ-Tocopherol	6.590 ± 0.030	1.070 ± 0.040	<0.001
δ-Tocopherol	0.280 ± 0.010	0.016 ± 0.002	<0.001
Total Tocopherols	6.956 ± 0.020	1.183 ± 0.040	<0.001
Sugar composition (g/100 g dw)			
Fructose	0.20 ± 0.01	0.34 ± 0.01	0.022
Glucose	0.21 ± 0.01	0.19 ± 0.01	0.178
Sucrose	1.97 ± 0.04	2.90 ± 0.10	<0.001
Trehalose	0.26 ± 0.01	0.25 ± 0.01	0.015
Total Sugars	2.64 ± 0.10	3.68 ± 0.10	<0.001
Organic acids composition (g/100 g dw)			
Oxalic acid	tr	0.006 ± 0.001	-
Malic acid	tr	tr	-
Total organic acids	-	0.006 ± 0.001	-

tr—traces. Significant differences (*p* < 0.001) between samples were determined according to Student’s *t*-test.

**Table 3:** Fatty acids profile (%) in pumpkin seeds, seed cake and seed oils (mean ± SD).

Chemical Structure	Compound Name	Seeds	Seed Cake	Seed Oil
C6:0	caproic acid	0.015 ± 0.001 *	0.168 ± 0.006 *	nd
C8:0	caprylic acid	0.002 ± 0.001 *	0.022 ± 0.001 *	nd
C10:0	capric acid	0.006 ± 0.001 *	0.011 ± 0.001 *	nd
C12:0	lauric acid	0.020 ± 0.001 *	0.039 ± 0.001 *	nd
C14:0	myristic acid	0.117 ± 0.004 c	0.230 ± 0.006 a	0.139 ± 0.006 b
C15:0	pentadecylic acid	0.020 ± 0.001 *	0.043 ± 0.001 *	nd
C16:0	palmitic acid	12.200 ± 0.040 b	13.987 ± 0.400a	14.306 ± 0.320 a
C16:1	palmitoleic acid	0.119 ± 0.004 c	0.162 ± 0.001 a	0.146 ± 0.001 b
C17:0	margaric acid	0.094 ± 0.004 b	0.092 ± 0.003 b	0.104 ± 0.004 a
C18:0	stearic acid	4.830 ± 0.080 c	5.460 ± 0.020 b	6.216 ± 0.020 a
C18:1n9c+t	oleic acid	37.027 ± 0.100 a	36.270 ± 0.020 a	21.950 ± 0.030 b
C18:2n6c	linoleic acid	43.890 ± 0.010 b	41.500 ± 0.300 c	55.460 ± 0.200 a
C18:3n3	α-linolenic acid	0.242 ± 0.004 c	0.585 ± 0.001 a	0.330 ± 0.030 b
C20:0	arachidic acid	0.359 ± 0.004 c	0.400 ± 0.002 a	0.394 ± 0.004 b
C20:1	gondoic acid	0.192 ± 0.001 b	0.242 ± 0.005 a	0.114 ± 0.001 c
C20:3n3 + C21:0	heneicosylic and eicosatrienoic acid	0.270 ± 0.010 *	0.154 ± 0.005 *	nd
C20:5n3	eicosapentaenoic acid	0.110 ± 0.010 *	0.063 ± 0.001 *	nd
C22:0	behenic acid	0.294 ± 0.009 b	0.430 ± 0.020 a	0.155 ± 0.003 c
C22:1n9	docosenoic acid	0.048 ± 0.003 *	0.016 ± 0.001 *	nd
C22:2	docosadienoic acid	nd	nd	0.220 ± 0.020
C23:0	tricosylic acid	0.027 ± 0.001 c	0.064 ± 0.001 b	0.466 ± 0.040 a
C24:0	lignoceric acid	0.118 ± 0.003 *	0.062 ± 0.001 *	nd
Total SFA (% of total FA)	saturated fatty acids	18.102 ± 0.030 c	21.048 ± 0.300 b	21.780 ± 0.300 a
Total MUFA (% of total FA)	monounsaturated fatty acids	37.412 ± 0.100 a	36.690 ± 0.010 a	22.210 ± 0.030 b
Total PUFA (% of total FA)	polyunsaturated fatty acids	44.486 ± 0.090 b	42.262 ± 0.300 c	56.010 ± 0.200 a

nd—not detected. Means in the same row followed by different Latin letters are significantly different based on Tukey’s HSD test at *p* = 0.05. * The asterisk symbol indicates significant differences (*p* < 0.001) between the two samples of the same row based on Student’s *t*-test.

**Table 4:** Hepatotoxic and anti-proliferative activity of the examined seeds, seed cake and seed oils extracts (GI_50_ values μg/mL).

	Hepatotoxicity (GI_50_ µg/mL)	Antiproliferative Activity (GI_50_ μg/mL)			
	PLP2 (Porcine Liver Primary Cell Line)	HeLa (Cervical Carcinoma)	HepG2 (Hepatocellular Carcinoma)	MCF-7 (Breast Carcinoma)	NCI-H460 (Non-Small Cell Lung Cancer)
Seeds	non-toxic *	non-toxic	non-toxic	non-toxic	non-toxic
Seed cake	non-toxic	non-toxic	non-toxic	non-toxic	non-toxic
Seed oils	non-toxic	327 ± 15	non-toxic	non-toxic	non-toxic

GI_50_ values refer to the concentration of sample which is responsible for 50% inhibition of growth in the studied cell lines. GI_50_ values for Ellipticine (positive control): 3 µg/mL (PLP2), 1.0 µg/mL (MCF-7), 1.0 µg/mL (NCI-H460), 2.0 µg/mL (HeLa), 1.0 µg/mL (HepG2) and 3.2 ± 0.7 μg/mL (PLP2). * non-toxic: GI_50_ > 400 μg/mL.

**Table 5:** Antibacterial activity of pumpkin seed oils extracts (MIC and MBC, mg/mL).

Substance	*Staphylococcus* *aureus* (ATCC 11632)		*Bacillus cereus* (Food Isolate)		*Micrococcus flavus* (ATCC 10240)		*Enterobacter cloacae* (ATCC 35030)		*Salmonella* Typhimurium (ATCC 13311)		*Escherichia coli* (ATCC 25922)	
	MIC	MBC	MIC	MBC	MIC	MBC	MIC	MBC	MIC	MBC	MIC	MBC
Seed oil	2.0	4.0	1.0	2.0	1.0	2.0	1.0	2.0	1.0	2.0	1.0	2.0
E211	4.0	4.0	0.5	0.5	1.0	2.0	2.0	4.0	1.0	2.0	1.0	2.0
E224	1.0	1.0	2.0	4.0	1.0	2.0	0.5	0.5	1.0	1.0	0.5	1.0

E211 and E244 were used as positive controls.

**Table 6:** Antifungal activity of pumpkin seed oils extracts (MIC and MFC, mg/mL).

Substance	*Aspergillus* *fumigatus* (ATCC 9197)		*Aspergillus* *versicolor* (ATCC 11730)		*Aspergillus niger* (ATCC 6275)		*Penicillium* *funiculosum* (ATCC 36839)		*Penicillium* *verrucosum* var. Cyclopium (Food Isolate)		*Trichoderma viride* (IAM 5061)	
	MIC	MFC	MIC	MFC	MIC	MFC	MIC	MFC	MIC	MFC	MIC	MFC
Seed oil	4.0	8.0	1.0	2.0	1.0	2.0	0.5	1.0	0.5	1.0	0.5	1.0
E211	1.0	2.0	2.0	4.0	1.0	2.0	1.0	2.0	2.0	4.0	1.0	2.0
E224	1.0	1.0	1.0	1.0	1.0	1.0	0.5	0.5	1.0	1.0	0.5	0.5

E211 and E244 were used as positive controls.

## Data Availability

The original contributions presented in the study are included in the article/Supplementary Materials, further inquiries can be directed to the corresponding authors.

## Supplementary Materials

The following supporting information can be downloaded at: https://www.mdpi.com/article/10.3390/plants13172395/s1. Figure S1: Tocopherol profile of the studied pumpkin seeds; 1- Mobile phase (MP), 2- α-Tocopherol, 3- β-Tocopherol, 4- γ-Tocopherol, 5- δ-Tocopherol, 6- Tocol (IS); Figure S2: Sugars profile of the studied pumpkin seed cake. 1- Mobile phase (M.P.), 2- Fructose, 3- Glucose, 4- Trehalose, 5- Melezitose (IS), Figure S3: Fatty acids profile of the studied pumpkin seeds. 1- C6:0, 2- C8:0, 3- C10:0, 4- C12:0, 5- C14:0, 6- C15:0, 7- C16:0, 8- C16:1, 9- C17:0, 10- C18:0, 11-C18:1n9c+t, 12- C18:2n6c, 13- C18:3n3, 14- C20:0, 15- C20:1, 16- C20:3n3 + C21:0, 17- C20:5n3, 18- C22:0, 19- C22:1n9, 20- C23:0, 21-C24:0; Figure S4: Fatty acids profile of the studied pumpkin seed oils. 1- C14:0, 2- C16:0, 3- C16:1, 4- C17:0, 5- C18:0, 6-C18:1n9c+t, 7- C18:2n6c, 8- C18:3n3, 9- C20:0, 10- C20:1, 11- C22:0, 12- C22:2, 13- C23:0.
