# Supplementary material for: Biochemical Composition of Pumpkin Seeds and Seed By-Products
Source: Plants (Basel). 2024 Aug 27;13(17):2395. doi: 10.3390/plants13172395 (PMC11397235; doi:10.3390/plants13172395)
Supplement: Supplementary file 1 [file plants-13-02395-s001.zip › plants-3160522-supplementary.pdf]

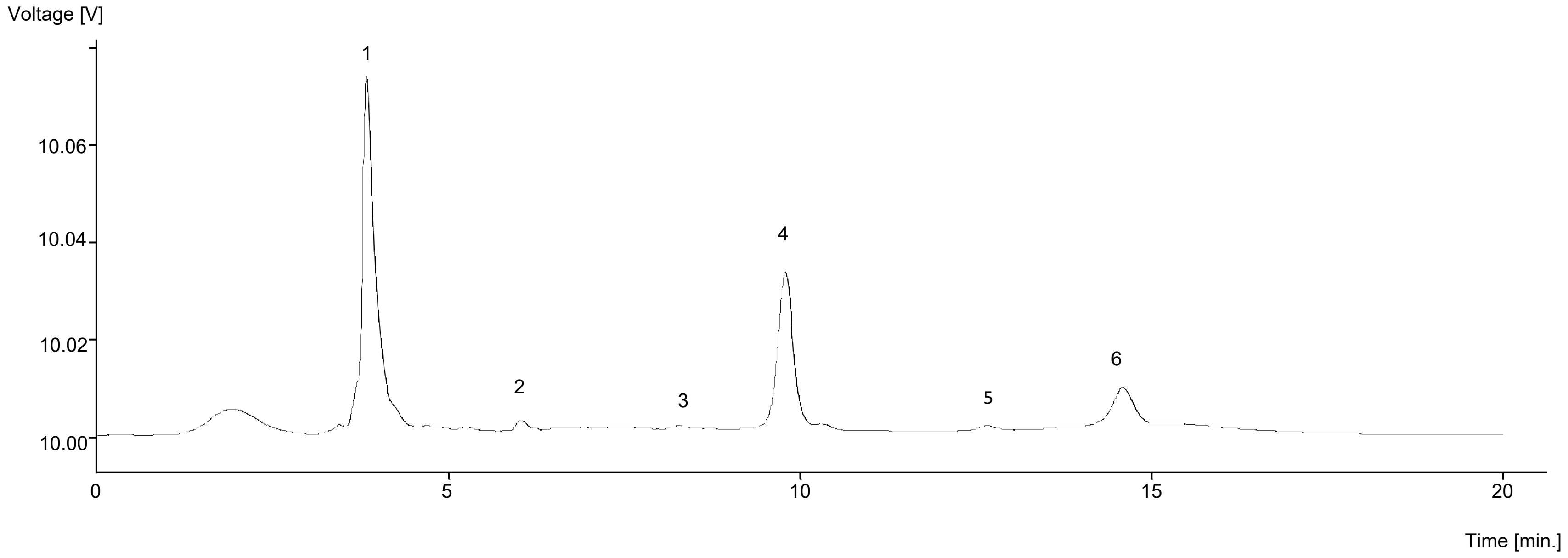

Figure S1. Tocopherol profile of the studied pumpkin seeds; 1- Mobile phase (MP), 2- α-Tocopherol, 3- β-Tocopherol, 4- γ-Tocopherol, 5- δ-Tocopherol, 6- Tocol (IS).

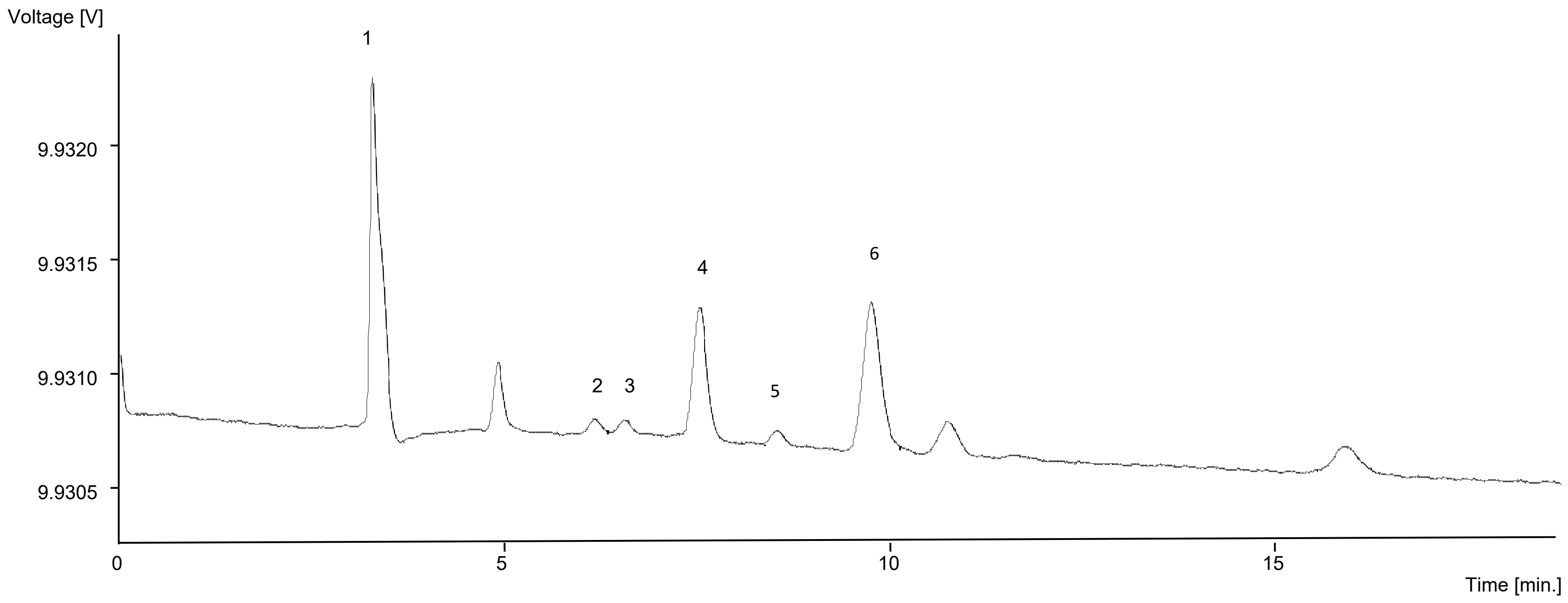

Figure S2. Sugars profile of the studied pumpkin seed cake. 1- Mobile phase (M.P.), 2- Fructose, 3- Glucose, 4- Trehalose, 5- Melezitose (IS).

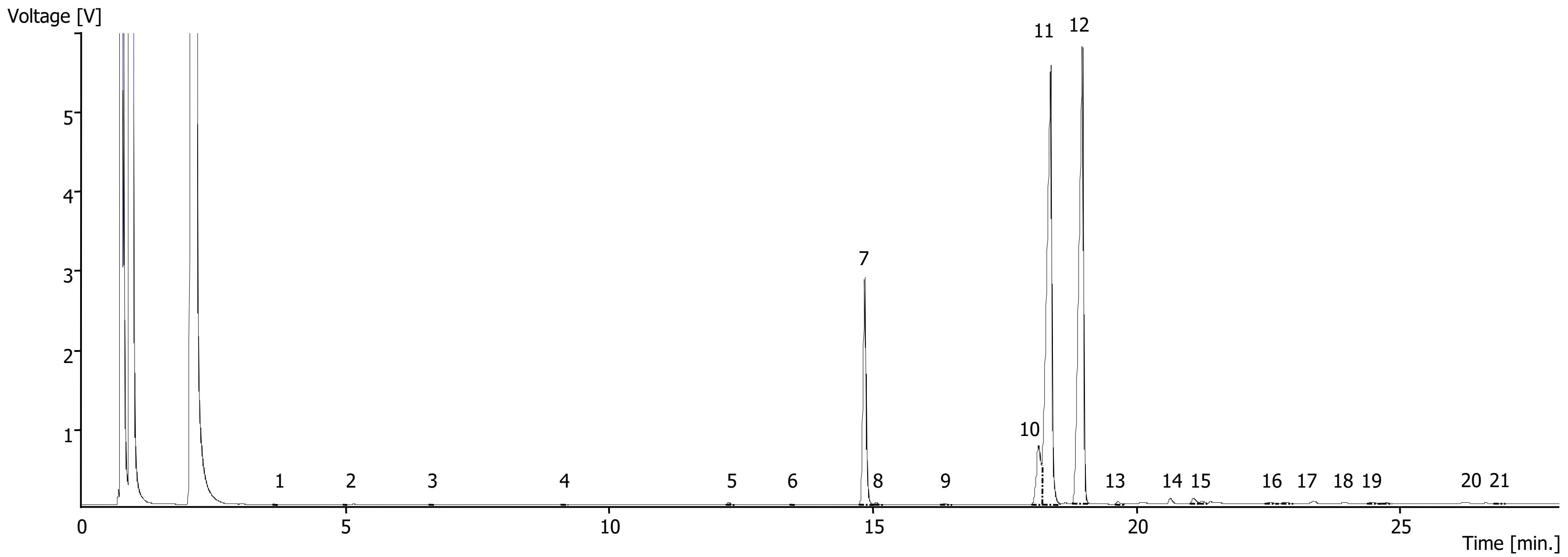

Figure S3. Fatty acids profile of the studied pumpkin seeds. 1- C6:0, 2- C8:0, 3- C10:0, 4- C12:0, 5- C14:0, 6- C15:0, 7- C16:0, 8- C16:1, 9- C17:0, 10- C18:0, 11-C18:1n9c+t, 12- C18:2n6c, 13- C18:3n3, 14- C20:0, 15- C20:1, 16- C20:3n3+C21:0, 17- C20:5n3, 18- C22:0, 19- C22:1n9, 20- C23:0, 21-C24:0.

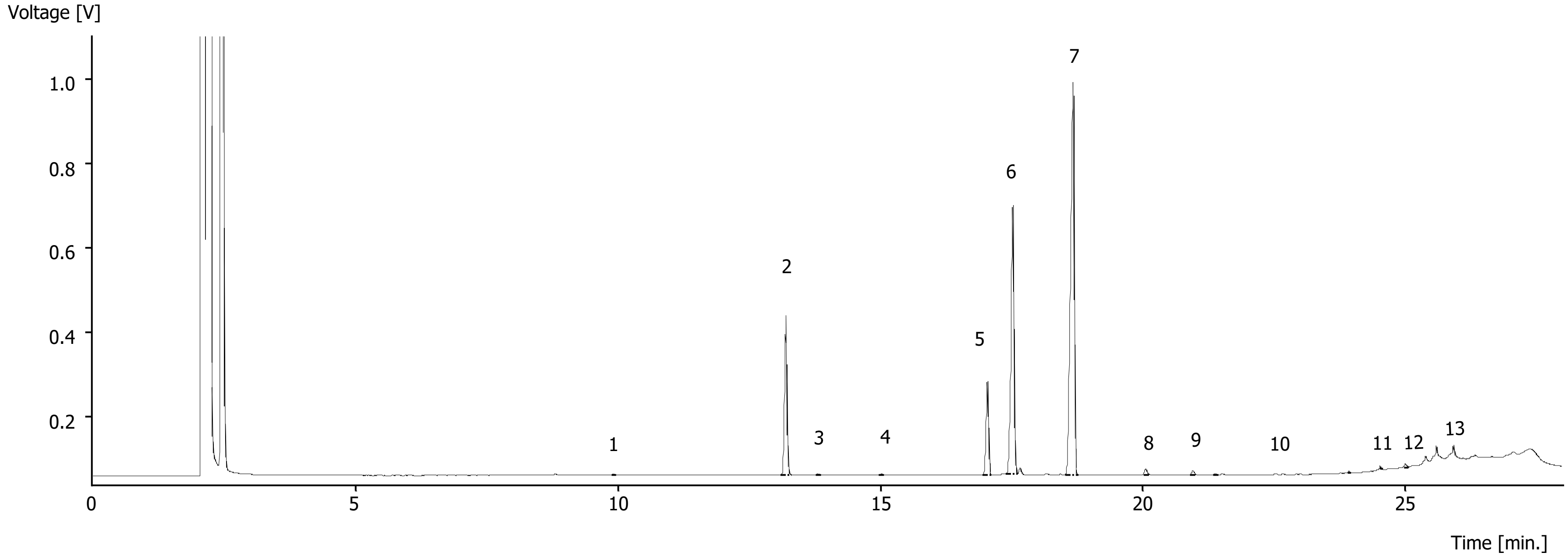

Figure S4. Fatty acids profile of the studied pumpkin seed oils. 1- C14:0, 2- C16:0, 3- C16:1, 4- C17:0, 5- C18:0, 6-C18:1n9c+t, 7- C18:2n6c, 8- C18:3n3, 9- C20:0, 10- C20:1, 11- C22:0, 12- C22:2, 13- C23:0
